# Unique regulation of TiO_2_ nanoporous topography on macrophage polarization via MSC-derived exosomes

**DOI:** 10.1093/rb/rbad012

**Published:** 2023-02-17

**Authors:** Jinjin Wang, Yazheng Wang, Yi Li, Yide He, Wen Song, Qintao Wang, Yumei Zhang, Chenyang He

**Affiliations:** State Key Laboratory of Military Stomatology & National Clinical Research Center for Oral Diseases & Shaanxi Engineering Research Center for Dental Materials and Advanced Manufacture, Department of Periodontology, School of Stomatology, The Fourth Military Medical University, Xi’an, Shannxi Province 710032, China; State Key Laboratory of Military Stomatology & National Clinical Research Center for Oral Diseases & Shaanxi Engineering Research Center for Dental Materials and Advanced Manufacture, Department of Periodontology, School of Stomatology, The Fourth Military Medical University, Xi’an, Shannxi Province 710032, China; State Key Laboratory of Military Stomatology & National Clinical Research Center for Oral Diseases & Shaanxi Key Laboratory of Stomatology, Department of Prosthodontics, School of Stomatology, The Fourth Military Medical University, Xi’an, Shannxi Province 710032, China; State Key Laboratory of Military Stomatology & National Clinical Research Center for Oral Diseases & Shaanxi Key Laboratory of Stomatology, Department of Prosthodontics, School of Stomatology, The Fourth Military Medical University, Xi’an, Shannxi Province 710032, China; State Key Laboratory of Military Stomatology & National Clinical Research Center for Oral Diseases & Shaanxi Key Laboratory of Stomatology, Department of Prosthodontics, School of Stomatology, The Fourth Military Medical University, Xi’an, Shannxi Province 710032, China; State Key Laboratory of Military Stomatology & National Clinical Research Center for Oral Diseases & Shaanxi Engineering Research Center for Dental Materials and Advanced Manufacture, Department of Periodontology, School of Stomatology, The Fourth Military Medical University, Xi’an, Shannxi Province 710032, China; State Key Laboratory of Military Stomatology & National Clinical Research Center for Oral Diseases & Shaanxi Key Laboratory of Stomatology, Department of Prosthodontics, School of Stomatology, The Fourth Military Medical University, Xi’an, Shannxi Province 710032, China; Department of Surgical Oncology, The Second Affiliated Hospital of Xi’an Jiaotong University, Xi’an, Shannxi Province 710004, China

**Keywords:** nanoporous topography, bone marrow mesenchymal stem cell, exosome, immunomodulatory effect, macrophage

## Abstract

The comprehensive recognition of communications between bone marrow mesenchymal stem cells (bm-MSCs) and macrophages in the peri-implant microenvironment is crucial for implantation prognosis. Our previous studies have clarified the indirect influence of Ti surface topography in the osteogenic differentiation of bm-MSCs through modulating macrophage polarization. However, cell communication is commutative and multi-directional. As the immune regulatory properties of MSCs have become increasingly prominent, whether bm-MSCs could also play an immunomodulatory role on macrophages under the influence of Ti surface topography is unclear. To further illuminate the communications between bm-MSCs and macrophages, the bm-MSCs inoculated on Ti with nanoporous topography were indirectly co-cultured with macrophages, and by blocking exosome secretion or extracting the purified exosomes to induce independently, we bidirectionally confirmed that under the influence of TiO_2_ nanoporous topography with 80–100 nm tube diameters, bm-MSCs can exert immunomodulatory effects through exosome-mediated paracrine actions and induce M1 polarization of macrophages, adversely affecting the osteogenic microenvironment around the implant. This finding provides a reference for the optimal design of the implant surface topography for inducing better bone regeneration.

## Graphical Abstract

**Figure:**
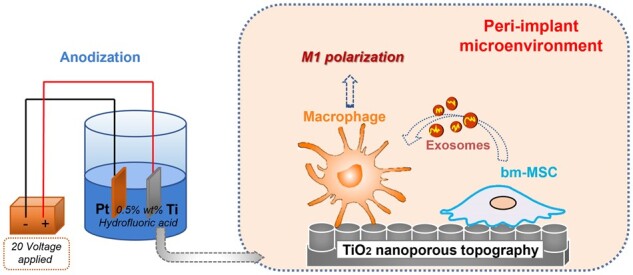

## Introduction

Dental implants have been widely used in clinics, which has solved the problem of dentition defects to a certain extent. However, given adverse reactions such as insufficient initial stability and the risk of peri-implantitis, there are significant challenges in achieving fast and stable osseointegration of the implants [1]. A comprehensive understanding of the cellular response in the peri-implant microenvironment is essential to improve prognosis.

After implantation, bone marrow mesenchymal stem cells (bm-MSCs) around the implant bed and in the distal bone marrow migrate to the implant through a migration and homing mechanism, which is an important cellular basis for early osseointegration [2]. Many studies have indicated that the physical topography modification of titanium (Ti) with various dimensions can significantly promote the osteogenic differentiation of bm-MSCs *in vitro* [3, 4]. Additionally, our previous research confirmed that the micro-nano topography of 80–100 nm diameter TiO_2_ nanotubes has a significant promoting effect on the osteogenic differentiation of bm-MSCs [5]. However, *in vivo* application of a variety of bone implantation materials fails to achieve ideal osseointegration [6], including TiO_2_ with a nanoporous topography, which may not achieve as excellent as *in vitro* osteogenic effect when applied *in vivo* [5]. The reason may be that the innate immune regulation of the implanted host to the materials plays different regulatory roles in the process of osseointegration [5–7].

As the key member of innate immune response, macrophage is significantly involved in the regulation process of the local tissue inflammatory response, and its different polarization directions determine the corresponding functional states. M1 macrophages secrete pro-inflammatory cytokines, such as interleukin (IL)-6, interferon-γ (IFN-γ), tumor necrosis factor-α (TNF-α) and inducer nitric oxide synthase (iNOS), whereas M2 macrophages attenuate inflammatory responses and promote wound healing process by secreting multitudinous anti-inflammatory cytokines, such as IL-4, IL-10 and arginase-1 (Arg-1) [8]. Over the past 10 years, many researchers have paid attention to the direct effect of biomaterials on macrophage polarization [7, 9, 10]. Moreover, in-depth studies have been performed on the effects of biomaterials on the osteogenic differentiation of bm-MSCs by regulating macrophage polarization [5]. In the microenvironment around the implant, however, cells can receive all types of biological signals through multiple cues [11], including cell-to-cell communications. In addition to be directly regulated by implant properties, macrophage polarization states could also be influenced by the signal exchanges between macrophages and other cells, such as the large number of bm-MSCs that are recruited [12, 13].

Apart from excellent self-renewal and multi-directional differentiation potential ability, MSCs have powerful immunomodulatory properties as well [14]. The immunomodulatory response of MSCs is plastic and can play completely different roles according to the local environment [15]. As the key MSCs in peri-implant microenvironment, we speculate that after implantation, bm-MSCs not only achieve osteogenic differentiation but also have significant immunomodulatory effects under the influence of the implant. Under this circumstance, what happens to the immune function of macrophages is unclear.

Extracellular vesicles (EVs) are crucial transmitters in the involvement of bm-MSCs in the innate and adaptive immune responses. Numerous studies have indicated that MSC-EV mediated intercellular communications could regulate immune cells, including T/B lymphocytes, natural killer cells, dendritic cells and macrophages [16]. In various EVs, exosome-mediated intercellular signaling has attracted considerable attention. Exosomes are small vesicles secreted by cells with diameters of 30–150 nm, which contain important molecular information such as mRNA, miRNA and protein, and can participate in the regulation of the immune response, inflammation, angiogenesis and other physiological processes [17, 18]. Exosome is considered as a new route of intercellular communication and contributes to maintaining tissue homeostasis. Studies have confirmed that MSCs-derived exosomes play a dominant role in regulating the physiological functions of macrophages and are important for the process of MSCs regulating tissue homeostasis [19–21]. Thus, bm-MSCs are likely to regulate macrophage functions mediated by exosomes under the influence of the surface topography of bone-implant materials.

To obtain a comprehensive understanding of the immunomodulatory effects of bm-MSCs on macrophages, and to clarify the interactions among bone-implant materials, bm-MSCs and macrophages in the peri-implant microenvironment, on the basis of our previous studies about the immediate effects of implant surface topography on physiological functions of bm-MSCs and macrophages, this study further examines the immunomodulatory effects of bm-MSCs mediated by exosomes on macrophage polarization under the influence of implant surface topography, to provide guidance for optimal surface design of bone-implant materials for better osseointegration.

## Materials and methods

### Preparation and characterization of Ti specimens

Commercially pure Ti plates (circular, 15 mm in diameter and 1 mm thick; column-shaped, 1 mm in diameter and 2 mm long) were provided by the Northwest Institute for Nonferrous Metal Research, Xi’an, China and were sequentially wet-polished with SiC sandpaper (400–7000 mesh) to obtain polished Ti specimens (PT). Subsequently, Ti specimens with nano-topography (NT) were fabricated via anodization in accordance with our previous reports [5]. The anodizing electrolyte comprising 372 ml of deionized water, 23 ml of 85% phosphate and 5 ml of hydrofluoric acid. The oxidation condition is a voltage of 20 V for 1 h. The fabricated topography was examined using field-emission scanning electron microscopy (FE-SEM, Hitachi, Japan), and the roughness was examined using atomic force microscopy (AFM, Shimadzu, Japan), and the hydrophilicity was assessed using a DSA1 System (Kruss, Germany). The fabricated specimens were sequentially cleaned in acetone, absolute ethanol and deionized water using ultrasonic cleaner. Then the cleaned specimens were immersed in 75% ethanol for 6–8 h and ultraviolet radiated for at least 30 min for sterilization.

### Effects of Ti specimens with nanoporous topography on osteogenesis *in vitro* and *in vivo*

#### Cell culture

The whole bone marrow culture of long bone was used to isolate bm-MSCs. The femurs and tibias of 6–8 weeks old C57BL/6J mice were selected. The typical procedure is the same as the previous study [5]. None-adherent cells were removed by frequent medium change during 72 h. After 9–12 days, the clustered adherent cells were considered as bm-MSCs, and they were passaged and inoculated onto blank plates (negative control, NC) and Ti specimens with different surface topographies. After 3 days of incubation, the culture supernatant was collected and centrifuged at 12 000 rpm for 10 min. Subsequently, the supernatant of each group was diluted in a ratio of 1:1 in the complete culture medium of macrophages (Roswell Park Memorial Institute, RPMI-1640, Hyclone, USA), and the resulting media were denoted as bm-MSCs conditioned medium (CM) of NC, PT and NT.

#### Osteogenic differentiation of bm-MSCs on Ti specimens with nanoporous topography

bm-MSCs were passaged and inoculated onto blank plates and Ti specimens with different surface topographies in 24-well plates (the inoculation density: 1.5 × 10^5^ cells/well) under the condition of osteoinductive medium (Cyagen Biosciences, Santa Clara, CA, USA). After 3 days of incubation, the total RNA of bm-MSCs was extracted using a Trizol reagent (Takara, Japan) and quantified using a Nanodrop 2000 (Thermo Fisher Scientific, USA). After reverse transcription, qRT-PCR was conducted to detect the mRNA expression of osteogenic-related genes [alkaline phosphatase (ALP), runt-related transcription factor 2 (Runx2), osteocalcin (OCN) and osteopontin (OPN)] according to the manufacturer’s protocols (Takara). The primers used in this study are presented in Table 1. After 7 and 21 days of incubation, an ALP activity assay and quantification of mineralization via alizarin red staining were conducted, as previously reported [5].

**Table 1.** Primers for quantitative reverse transcription polymerase chain reaction

Gene	Forward primer sequence (5′–3′)	Reverse primer sequence (3′–5′)
ALP	GACTGGTACTCGGATAACGA	TGCGGTTCCAGACATAGTGG
Runx2	ATGCTTCATTCGCCTCACAAA	GCACTCACTGACTCGGTTGG
OCN	TGCTTGTGACGAGCTATCAG	GAGGACAGGGAGGATCAAGT
OPN	AGCAAGAAACTCTTCCAAGCAA	GTGAGATTCGTCAGATTCATTCCG
iNOS	GGACCCAGTGCCCTGCTTT	CACCAAGCTCATGCGGCCT
IL-6	TAGTCCTTCCTACCCCAATTTCC	TTGGTCCTTAGCCACTCCTTC
TNF-α	CTTCTCCTTCCTGATCGTGG	GCTGGTTATCTCTCAG
Arg-1	CTCCAAGCCAAAGTCCTTAGAG	AGGAGCTGTCATTAGGGACATC
IL-4	ACAGGAGAAGGGACGCCAT	GAAGCCCTACAGACGAGCTCA
IL-10	GCTCTTACTGACTGGCATGAG	CGCAGCTCTAGGAGCATGTG
GAPDH	TGTGTCCGTCGTGGATCTGA	TTGCTGTTGAAGTCGCAGGAG

#### Osseointegration of Ti implant with nanoporous topography in mice

Sixteen C57BL/6J mice (male, 6–8 weeks, 20 g) were included and randomly distributed into two groups (PT and NT). Femur implantation was achieved using previously reported procedures [5]. The animal procedures were approved by the university research ethics committee of the Fourth Military Medical University and in accordance with the guidelines for the management and use of laboratory animals. The laboratory mice were sacrificed at 3 weeks, and the femur specimens were obtained. The femur specimens were fixed in 4% paraformaldehyde, then the osseointegration of implants was analyzed using a micro-computed tomography (micro-CT) scanner (YXLON International GmbH, Germany). Furthermore, the femur specimens were decalcified using ethylenediaminetetraacetic acid, and paraffin tissue sections were fabricated for hematoxylin & eosin (HE) and immunohistochemical (IHC) staining.

After the 3D scan reconstruction of femur specimens was completed, an area of 2 mm around the implant was selected as the region of interest (ROI), and the images were analyzed via VGStudio Max 2.2 (Volume Graphic, Germany). The new bone volume ratio (bone volume to total volume, BV/TV), trabecular thickness (TbTh), trabecular numbers (TbN) and trabecular separation (TbSp) of each specimen were determined and statistically analyzed. The infiltration of inflammatory cells and the expression level of M1 macrophage markers-iNOS (Abcam, UK, the dilution was 1:100) around the implant were detected via HE staining and IHC staining, respectively. The streptavidin-peroxidase method was used for IHC staining, in accordance with the manufacturer’s protocols (ZSGB-BIO, Beijing, China). Histological images of femur specimens were obtained using an optical microscope (Olympus, Japan) and analyzed via NIH Image J software.

### Effects of conditioned media of bm-MSCs cultured on Ti specimens with nanoporous topography on macrophage polarization

#### Cell culture

The RAW264.7 cell line was purchased from ATCC and cultured under conventional culture conditions. The purity of cells was detected by flow cytometry using F4/80 labeling. After 24 h of adherent culturing, the macrophage culture medium RPMI-1640 was replaced with the CM of bm-MSCs in each group (the preparation of CM is described in the previous ‘Cell culture’ section), and then continued to be cultured for another 3 days.

#### mRNA level of M1 macrophage markers

After 3 days of incubation, the total RNA of RAW264.7 was extracted, quantified and reversely transcribed (Takara) following the same procedures as previously described [5]. qRT-PCR was performed according to the manufacturer’s protocols (Takara). Primers used in the study are displayed in Table 1.

#### Protein level of M1 macrophage markers

The surface markers CCR7 (M1) and CD206 (M2) were examined via flow cytometry for evaluating different macrophage phenotypes. The specific procedure is the same as described in previous studies [5]. Anti-mouse CD16/32 (Biolegend, USA) was used to block the non-specific antigens. The antibodies for flow cytometry in this study included PE-conjugated CCR7 and PerCP-conjugated CD206 (Biolegend, USA), and the isotype controls were PE-conjugated Rat IgG2a, ĸ and PerCP-conjugated Rat IgG2a, ĸ (Biolegend, USA). The expression level of M1/M2 macrophage markers of all samples was analyzed using a flow cytometer (NovoCyte, ACEA Biosciences, USA) in triplicate. In addition, the surface markers IL-1β (M1) and CD163 (M2) were detected via western blot for further evaluating different macrophage phenotypes. The first antibodies for western blot were anti-IL-1β (ABclonal, Wuhan, China, the dilution was 1:1000), anti-CD163 (ABclonal, the dilution was 1:1000) and anti-glyceraldehyde-3-phosphate dehydrogenase (GAPDH) (ABclonal, the dilution was 1:4000).

### Effects of bm-MSC-derived exosomes on Ti specimens with nanoporous topography on macrophage polarization

#### Isolation, extraction, identification and inhibition of MSC-derived exosomes

bm-MSCs were inoculated on blank plates and Ti specimens with different surface topographies in 24-well plates (the inoculation density: 1.5 × 10^5^ cells/well). After 24 h of incubation, the serum without exosomes replaced the normal serum in the culture medium, and then bm-MSCs continued to be cultured for another 48 h. The cultured supernatant of bm-MSCs was collected from each group, and exosomes were extracted via differential centrifugation at 4°C. In general, the supernatant was centrifuged at 300×g/min for 10 min, 2000×g/min for 10 min and 16000×g/min for 30 min. Then, the supernatant was dumped, and the sediment was centrifuged in an overspeed centrifuge at 150 000×g/min for 70 min. The supernatant was dumped and then re-suspended in PBS and centrifuged at 150 000×g/min for 70 min. The groups were denoted as NC-Exo, PT-Exo and NT-Exo.

The morphology and particle-size distribution of the exosomes was characterized via transmission electron microscopy (TEM) and nanoparticle tracking analysis (NTA). The exosome surface proteins were lysed using radioimmunoprecipitation assay lysis buffer and quantified with a bicinchonininc acid assay kit (Beyotime, Shanghai, China). Proteins with different molecules were separated via 10% sodium dodecyl sulfate polyacrylamide gel electrophoresis (SDS-PAGE) and transferred onto polyvinylidene difluoride (PVDF) membranes. Following the block with 5% bovine serum albumin in Tris–HCl balanced salt solution with Tween-20 (TBST, 10 mM Tris–HCl pH 7.5, 150 mM NaCl, 0.1% Tween-20) for 1 h, the membranes were incubated in primary antibody diluent at 4°C overnight, and incubated with horseradish peroxidase-conjugated secondary antibodies (Cell Signaling Technology, USA) at the ambient temperature for 2 h. The first antibodies were anti-HSP90B1 (Cell Signaling Technology, the dilution was 1:1000), anti-TSG101 (Abcam, UK, the dilution was 1:1000), anti-CD9 (Abcam, the dilution was 1:1000) and anti-CD63 (Abcam, the dilution was 1:2000). The bands were visualized using an enhanced chemiluminescence reagent (Thermo Fisher Scientific). Subsequently, images were obtained via Tanon5500 (Shanghai, China) and analyzed using Image J software.

The bm-MSC-derived exosomes were labeled with PKH26 and added into the culture medium of macrophages for 3 h. Subsequently, the macrophage membrane was labeled with AF488, and the nucleus was labeled with Hochest 33342. The uptake of bm-MSC-derived exosomes by macrophages was detected under confocal laser microscopy.

The secretion of exosomes was inhibited by GW4869. As described previously, bm-MSCs were inoculated on the blank culture plates in 24-well plates (1.5 × 10^5^ cells/well). When the cells adhered to the plate, and the cells were fused to 90% after 24 h of incubation, GW4869 (5 μM) was added into the culture medium, and bm-MSCs were continued to be cultured for another 48 h. Then the GW4869-pretreated bm-MSCs were harvested and inoculated on the blank plates and Ti specimens with different surface topographies in 24-well plates (the inoculation density: 1.5 × 10^5^ cells/well) under the condition of complete medium culture. After 3 days of incubation, the culture supernatant of each group was collected and centrifuged as before. The conditioned media without exosomes were denoted as NC-G, PT-G and NT-G.

#### mRNA and protein level of M1 macrophage markers

The bm-MSC-derived exosomes from each group were added into the macrophage culture system at the final concentration of 20 μg/ml with PBS. Macrophages were stimulated with PBS only as a solvent control group. After 3 days of incubation, the expression of macrophage polarization markers was detected via qRT-PCR, flow cytometry and western blot (protocols similar to those described previously).

### Statistical analysis

Each experiment was independently performed three times. The GraphPad Prism 9.0 software was used for statistical analysis. Experimental data were presented as the mean ± standard deviation of samples (*x* ± *S*). Student’s *t* test was used for comparison between the two groups, and a one-way analysis of variance was used for three or more groups. The set test level α = 0.05 and bilateral *P* < 0.05 were considered to be statistically significant.

## Results

### Surface characteristics of TiO_2_ nanoporous topography

**Figure 1.**
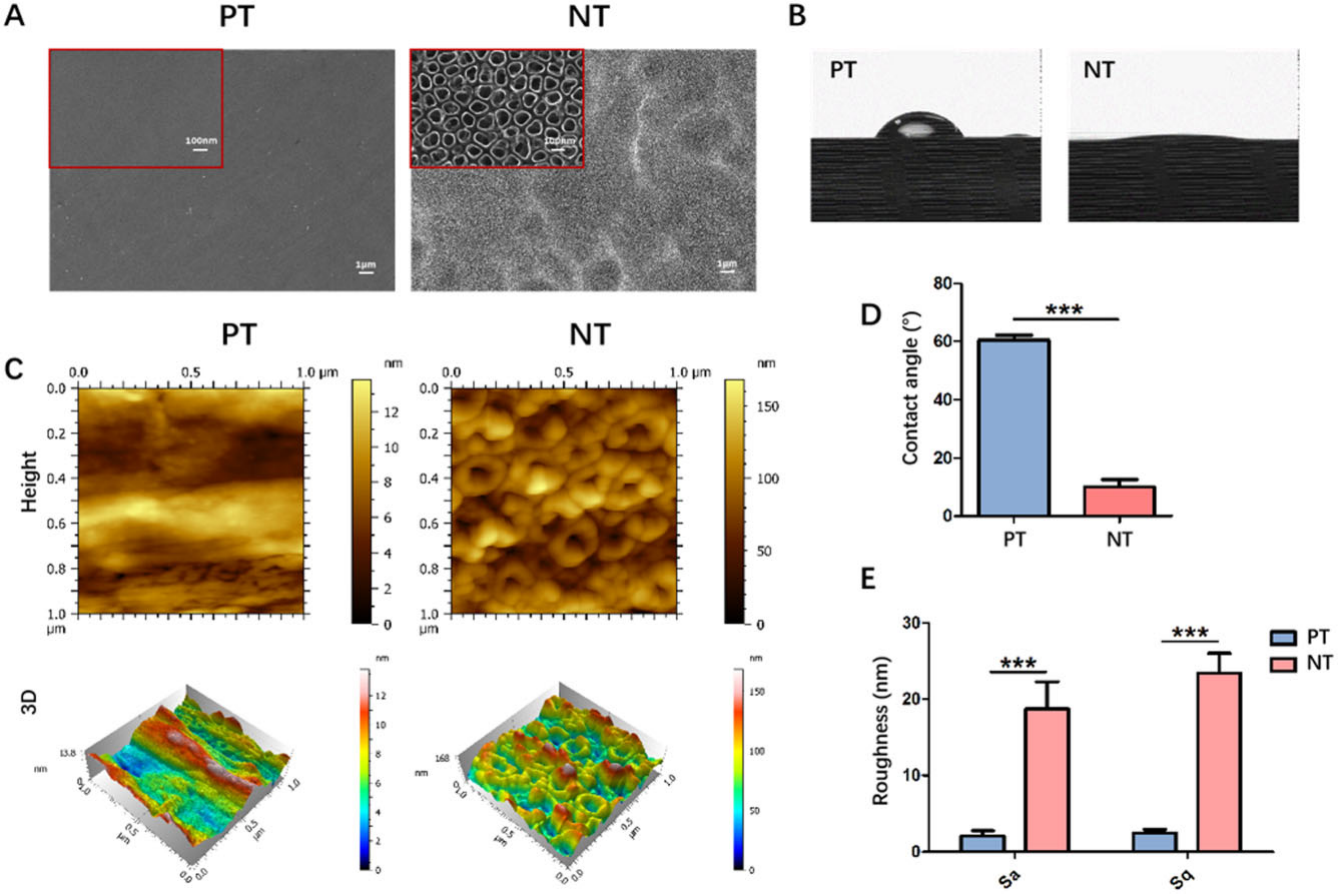
Characterization of Ti specimens. (**A**) Surface topographies were examined using FE-SEM. (**B**, **D**) The hydrophilicity of Ti specimens was evaluated using the water contact angles. (**C**, **E**) The surface roughness of Ti specimens was examined using AFM. PT, polished Ti surface; NT, nanoporous Ti surface. ***P < 0.001.

The surface topographies of both Ti specimens were characterized via FE-SEM. As shown in Fig. 1, the surface of PT was uniformly polished, whereas the tubular structure with a diameter of 80–100 nm was observed on NT surface (Fig. 1A). The roughness of both Ti specimens was evaluated via AFM. The surface roughness of NT was ∼10 times that of PT (Fig. 1C and E). The surface hydrophilicity of Ti specimens was reflected by the size of contact angle, which was ∼66° ± 1.75° for PT and 23° ± 3.12° for NT, indicating that the hydrophilicity of NT was better than that of PT (Fig. 1B and D).

### Osteogenic induction of nanoporous topography *in vitro* and *in vivo*

**Figure 2.**
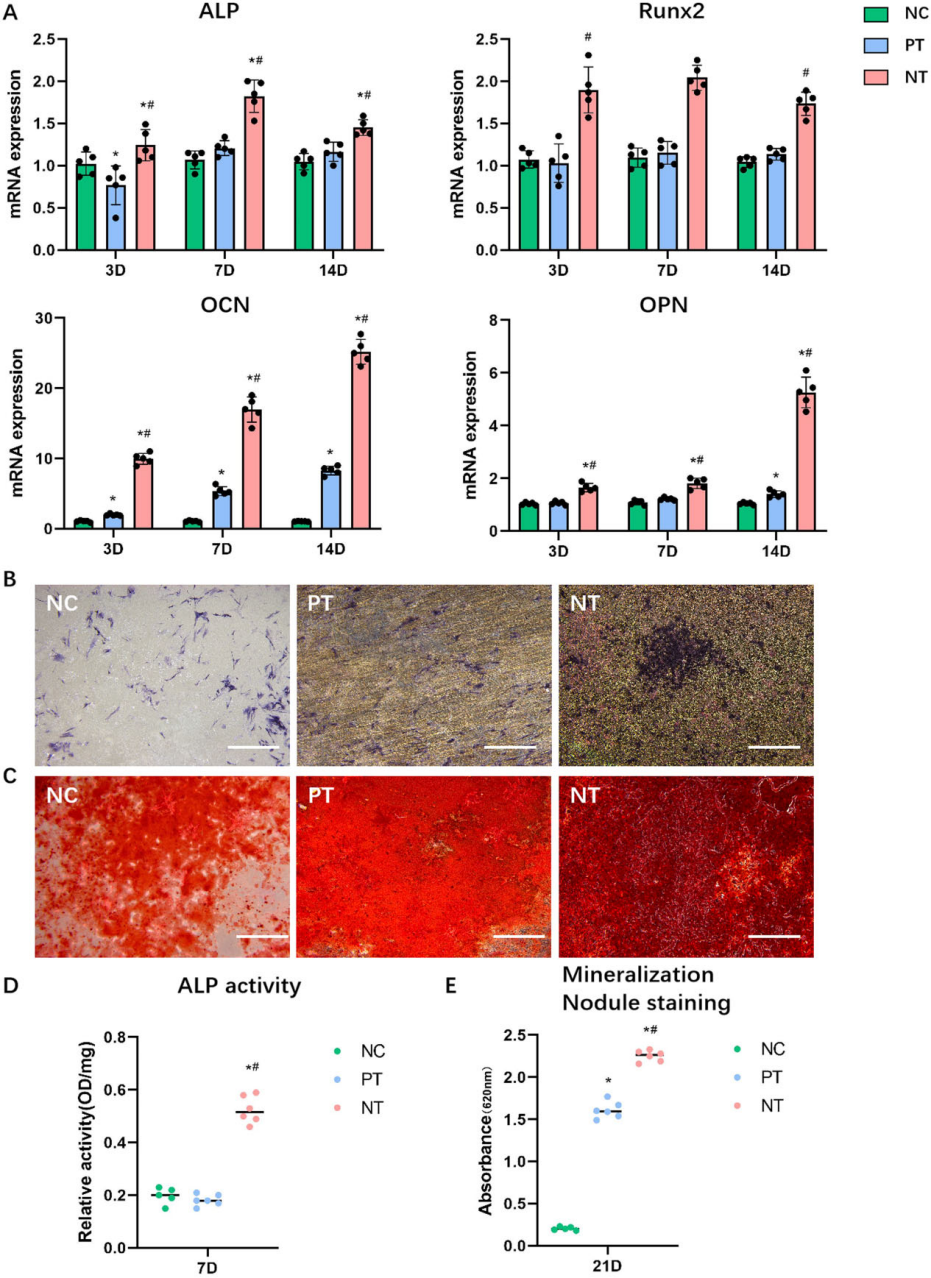
Osteogenic differentiation of mice bm-MSCs under the effects of different topographies *in vitro*. (**A**) qRT-PCR analysis of mRNA expression of ALP, Runx2, OCN and OPN in bm-MSCs after incubation for 3, 7 and 14 days. (**B**, **D**) ALP staining and ALP activity measurement after incubation for 7 days. (**C**, **E**) Alizarin red staining and semi-quantitative analysis after incubation for 21 days. NC, negative control; PT, polished Ti; NT, nanoporous Ti. *P < 0.01 vs NC, ^#^*P* < 0.01 vs PT. Scale bar = 100 μm.

The inductive effects of TiO_2_ nanoporous topography to the osteogenic differentiation of bm-MSCs was determined via qRT-PCR analysis, ALP staining and alizarin red staining. The mRNA expression of genes associated with osteogenesis (ALP, Runx2, OCN and OPN) was significantly enhanced in NT group compared with PT and NC in 3, 7 and 14 days (Fig. 2A). Additionally, the degree of ALP synthesis by bm-MSCs of NT was increased significantly compared with those of NC and PT, while there was no significant difference between NC and PT (Fig. 2B and D). Consistently, after incubation for 21 days, the most mineralized nodules were observed in the NT group. Furthermore, the enhanced formation of mineralized nodules in PT compared with NC was observed (Fig. 2C and E).

**Figure 3.**
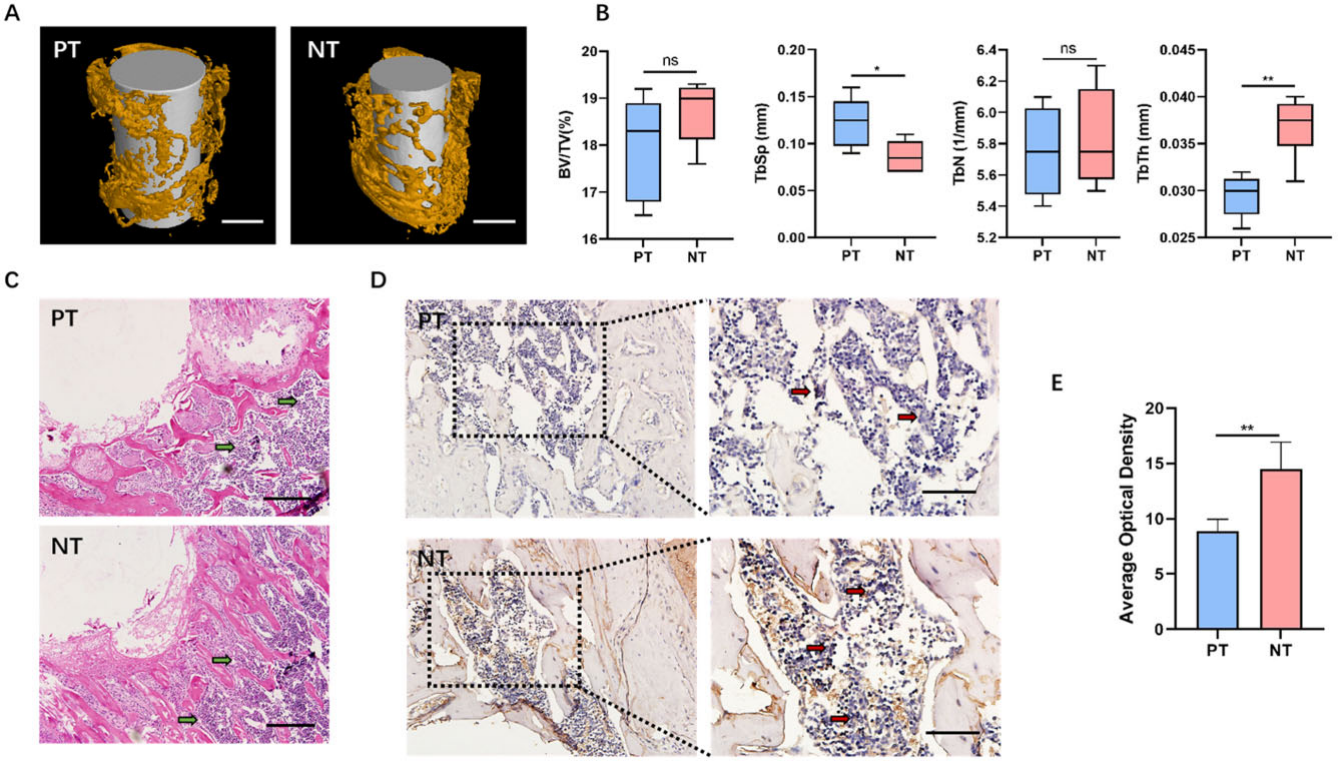
Osteogenesis of implants with different topographies in mice. (**A**) Micro-CT analysis of peri-implant osteogenesis, with a 2-mm area around the implant defined as the ROI. Scale bar = 500 μm. (**B**) Quantitative analysis of the bone volume fraction (BV/TV), trabecular separation (TbSp), trabecular number (TbN) and trabecular thickness (TbTh); (**C**) histological analysis of peri-implant inflammation via HE staining. The space in the upper left corner indicates the location of the implant. The green arrows indicate the infiltrating inflammatory cells. Scale bar = 200 μm. (**D**) Analysis of macrophage polarization around the implant via IHC staining. The red arrows indicate the iNOS-positive cells (M1 macrophages). scale bar = 100 μm. (**E**) The semi-quantitative analysis of IHC staining. *P < 0.05, **P < 0.01; ns, no significance.

However, the micro-CT results indicated that the bone around the implant was sparse, and no uniform and compact trabecular structure was observed in the PT or NT group. The results of 3D scan reconstruction analysis revealed that, except for TbTh and TbSp, other indicators of osteogenesis in the ROI in NT group was not significantly different from that in PT group (Fig. 3A and B). In the tissue sections of peri-implant femurs, a large number of infiltrating inflammatory cells was found in the target area for both PT and NT groups (Fig. 3C). Moreover, among the infiltrating inflammatory cells, numerous cells positively expressed iNOS, suggesting that macrophages in the peri-implant microenvironment tended to be M1-polarized (Fig. 3D). In addition, the number of M1 macrophages around the implant in NT group was even higher than that in PT group (Fig. 3E).

### Influence of bm-MSCs regulated by TiO_2_ nanoporous topography on macrophage polarization

**Figure 4.**
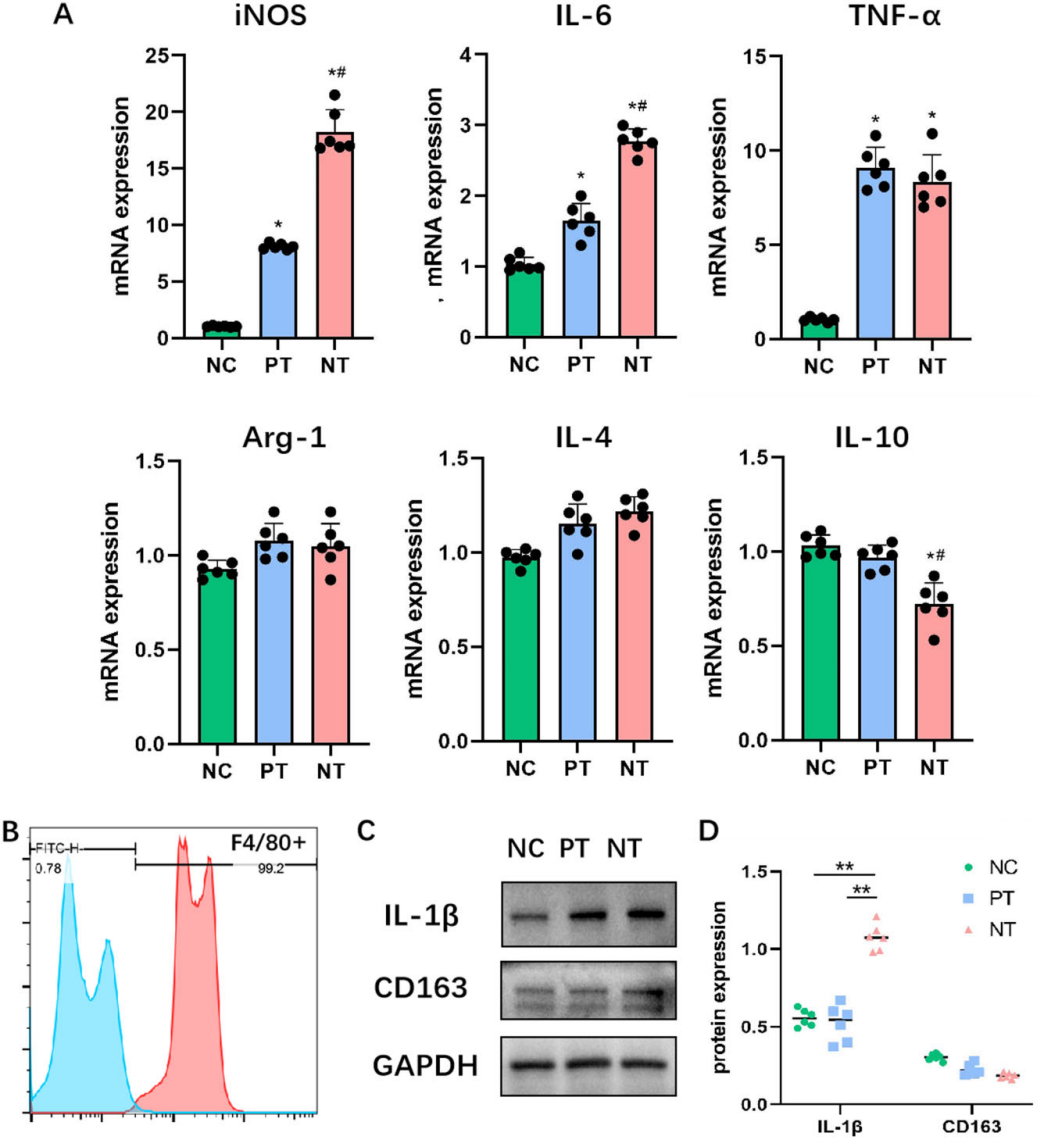
Effects of CM for bm-MSCs induced by the different surface topographies of Ti specimens on macrophage polarization. (**A**) qRT-PCR analysis of mRNA expression for M1/M2 macrophage markers (M1: iNOS, IL-6 and TNF-α; M2: Arg-1, IL-4 and IL-10). (**B**) Flow cytometry with F4/80 labeling for evaluating the purity of mouse macrophage lines RAW264.7. (**C**) Western blot analysis of protein expression for M1/M2 macrophage markers (M1: IL-1β; M2: CD163). (**D**) Semi-quantitative analysis of the protein expression level. (A) *P < 0.01 vs NC, ^#^*P* < 0.01 vs PT; (D) **P < 0.01.

To detect the effects of bm-MSCs cultured on either PT or NT on the polarization of macrophages, the latter were co-cultured indirectly using a CM of bm-MSCs on Ti surfaces with different topographies. First, the purity of the macrophage cell line RAW264.7 was identified with F4/80 labeling and was found to be >99% (Fig. 4B). Then, qRT-PCR analysis revealed that the conditioned media of bm-MSCs in both PT and NT groups were more prone to induce macrophages to overexpress iNOS, IL-6 and TNF-α compared with NC, which were mostly M1 polarization markers. In particular, the macrophages in NT group exhibited highly significant expression of iNOS and IL-6 among the three groups. In contrast, the macrophages in PT and NT groups did not manifest significantly different expressions of Arg-1 and IL-4 compared with NC; furthermore, the IL-10 expression was significantly lower in NT group than in the other groups (Fig. 4A). Western blot verified the higher protein expression of M1 macrophage marker IL-1β and lower protein expression of M2 macrophage marker CD163 in NT group (Fig. 4C and D).

### Influence of bm-MSCs-derived exosomes regulated by TiO_2_ nanoporous topography on macrophage polarization

**Figure 5.**
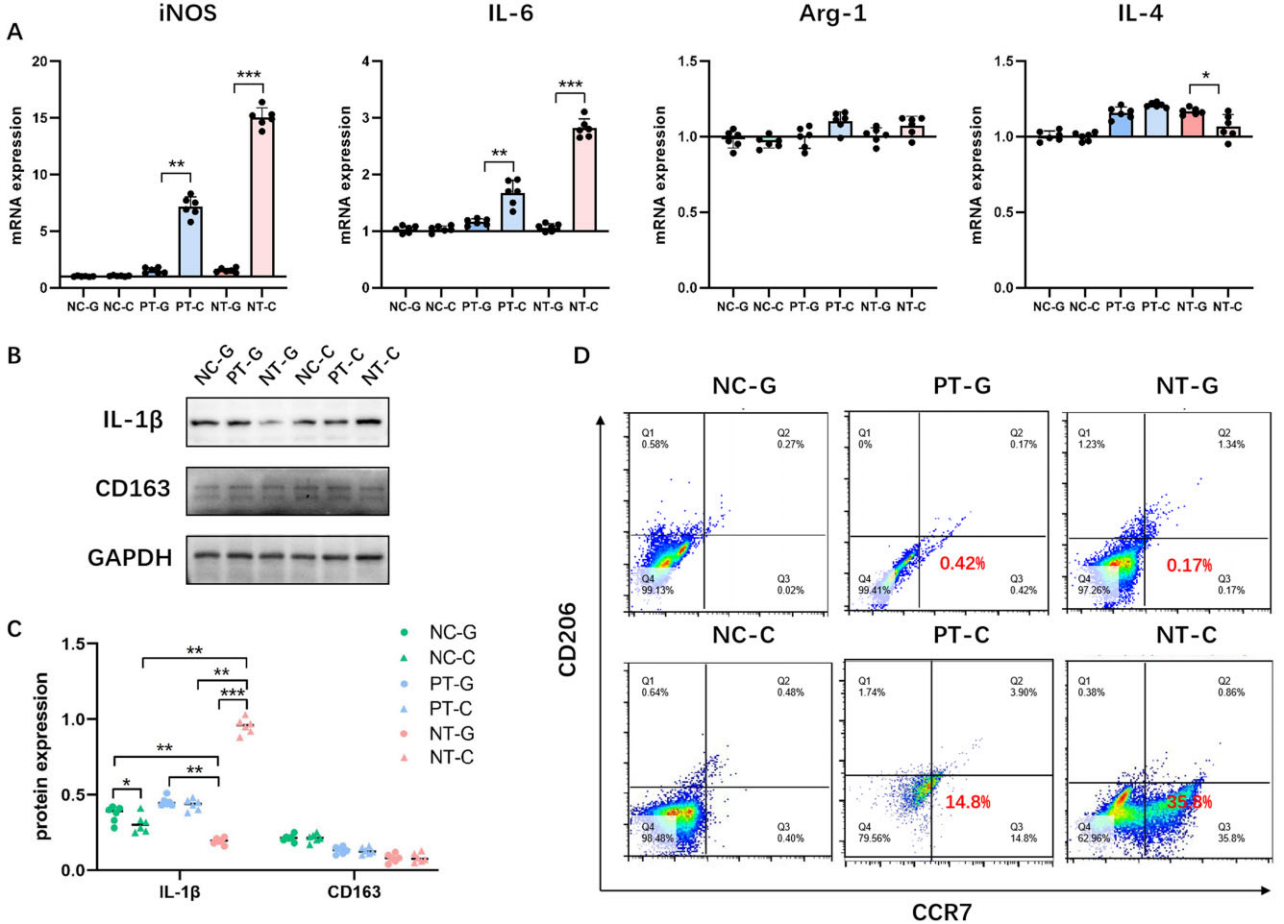
Effects of CM without exosomes for bm-MSCs induced by the different surface topographies of Ti specimens on macrophage polarization. The secretion of exosomes was blocked by GW4869 (or DMSO as control). (**A**) qRT-PCR analysis of mRNA expression of M1/M2 macrophage markers (M1: iNOS and IL-6; M2: Arg-1 and IL-4). (**B**) Western blot analysis of protein expression of the M1/M2 macrophage markers (M1: IL-1β; M2: CD163). (**C**) Semi-quantitative analysis of the protein expression level. (**D**) Flow cytometry analysis of M1/M2 macrophage markers (M1: CCR7; M2: CD206). NC-C, negative control with DMSO; PT-C, polished Ti with DMSO; NT-C, nanoporous Ti with DMSO; NC-G, negative control with GW4869; PT-G, polished Ti with GW4869; NT-G, nanoporous Ti with GW4869. *P < 0.05, **P < 0.01, ***P < 0.001.

Paracrine is an important pathway of immunomodulatory function of MSCs. To detect whether bm-MSCs-derived exosomes cultured on nanoporous Ti have a significant effect on macrophage M1 polarization, GW4869 was used to block the secretion of exosomes from bm-MSCs, then the CM without exosomes of bm-MSCs was used for indirect co-culturing with macrophages. qRT-PCR analysis revealed that after the exosomes from bm-MSCs were blocked, the CM in NT group induced the down-regulation of iNOS and IL-6, and the up-regulation of IL-4 in macrophages, while the expressions of Arg-1 hardly differed among the three groups (Fig. 5A). Western blot results revealed that the protein expression of M1 macrophage marker IL-1β in NT-G was significantly reduced compared with the other groups, while the protein expression of M2 macrophage marker CD163 was barely changed for both PT-G and NT-G (Fig. 5B and C). Similar results were obtained via flow cytometry; that is the CM of bm-MSCs for both PT-G and NT-G induced evidently reduced expression of M1 macrophage marker CCR7, while there was no significant difference in the expression of M2 macrophage marker CD206 among the three groups (Fig. 5D).

**Figure 6.**
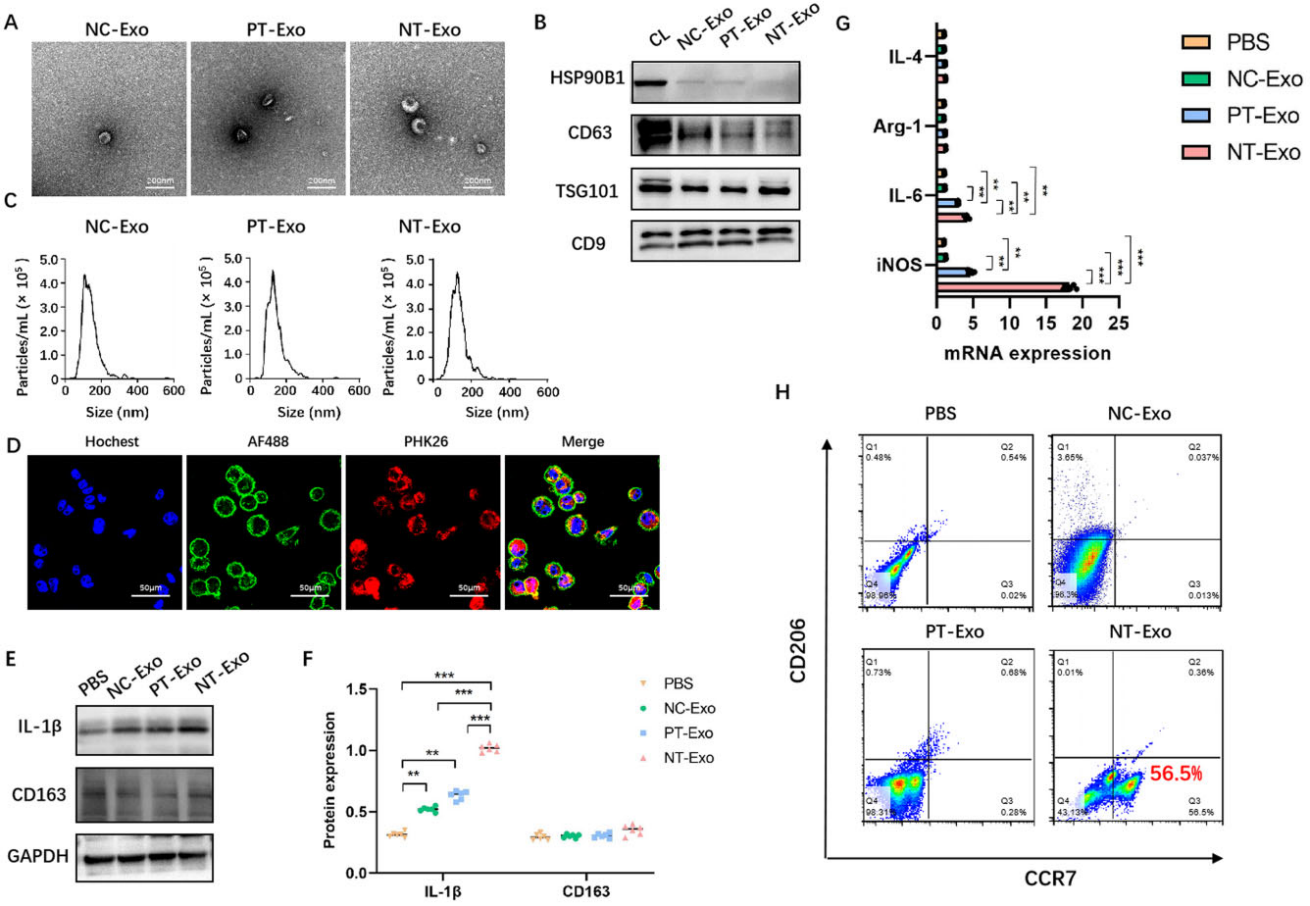
Identification of exosomes derived from bm-MSCs cultured on the surface of Ti with different topographies and the influence of bm-MSC-derived exosomes on macrophage polarization. (**A**) TEM observation; scale bar = 200 nm. (**B**) Western blot analysis of the expression level of exosome surface proteins. (**C**) NTA of exosome particle-size distribution. (**D**) Observation of exosome uptaken by macrophages. (**E**) Western blot analysis of the protein expression level of macrophage polarization markers induced by exosomes derived from bm-MSCs. (**F**) Semi-quantification of western blot analysis. (**G**) qRT-PCR analysis of the mRNA expression of macrophage polarization markers induced by exosomes derived from bm-MSCs. (**H**) Flow cytometry analysis of macrophage polarization marker expression induced by exosomes derived from bm-MSCs. CL, the total protein of bm-MSCs; PBS, solvent control for solubilizing exosomes; NC-Exo, exosomes from bm-MSCs on blank culture plates; PT-Exo, exosomes from bm-MSCs on Ti with polished surface; NT-Exo, exosomes from bm-MSCs on Ti with nanoporous topography. **P < 0.01, ***P < 0.001.

To further verify the role of exosomes derived from bm-MSCs in the regulation of macrophage polarization, we extracted and identified the exosomes from bm-MSCs via differential centrifugation. TEM indicated that the precipitations obtained via differential centrifugation from the culture supernatants of bm-MSCs in NC, PT and NT groups all had a double-layer membrane structure, which was cup-shaped or quasi-circular, with a diameter of <200 nm, conforming to the morphological characteristics of exosomes (Fig. 6A). The NTA results indicated that the peak particle size of exosomes from bm-MSCs in PT group was 127 nm, with a peak area of 90.4%, and that for NT group was 129.8 nm, with a peak area of 93.5%, both satisfying the standard for the particle-size distribution of exosomes (Fig. 6C). Western blot results indicated that bm-MSC-derived exosomes had negative expression of intracellular protein HSP90B1 and positive expression of transmembrane or lipid-binding-related extracellular proteins (CD9 and CD63) and cytoplasmic proteins (TSG101) (Fig. 6B). To determine whether macrophages can take in exosomes, PHK26 was used to label the exosomes derived from bm-MSCs; AF488 was used to label the cell membranes of macrophages, and Hochest 33342 was used to label the nuclei of macrophages. Using confocal laser microscopy, macrophages taking in exosomes were observed (Fig. 6D).

After characterization and identification, bm-MSC-derived exosomes in NC, PT and NT groups were added into the macrophage culture medium to clarify the regulatory effect of bm-MSC-derived exosomes on macrophage M1 polarization. PBS was used as a solvent control for solubilizing exosomes. qRT-PCR analysis revealed that the mRNA expression of iNOS and IL-6 in NT group was significantly elevated than that of the other groups, whereas the mRNA expression of M2 macrophage markers showed no significant difference. In addition, exosomes from PT group induced significantly higher expression of M1 macrophage markers compared with the PBS and NC groups (Fig. 6G). Western blot results suggested that the protein expression of IL-1β in NT group was significantly elevated, whereas the protein expressions of IL-1β in NC and PT groups were nearly identical and significantly higher than those of the PBS group. There was no significant difference in the expression of CD163 among the four groups (Fig. 6E and F). Flow cytometry results revealed that bm-MSC-derived exosomes in NT group promoted significantly higher expression of M1 macrophage marker CCR7 and rarely changed the expression of M2 macrophage marker CD206 compared with other groups (Fig. 6H). These results suggested that bm-MSC-derived exosomes induced by Ti with nanoporous topography could effectively induce the M1 polarization of macrophages.

## Discussion

A nano-topography with a biomimetic hierarchical structure was constructed on the surface of pure Ti to simulate the gradient structure of natural bone tissue, which can promote the osseointegration of implants. Our research group has been devoted to promoting osteogenesis of materials through physical modification of the Ti surface topography, and we found that TiO_2_ nanotube structure with different tube diameters was prone to promote osteogenic differentiation of MSCs *in vitro* [5, 7]. However, under *in vivo* conditions, the composition around the implant is complex with a diverse microenvironment. This may include osteogenic basic cells, for example, MSCs and various immune cells, among which macrophages related to the tissue inflammatory response are crucial for the prognosis of implant [2]. Upon implantation of biomaterials, MSCs and macrophages contribute jointly to the wound healing and the subsequent regeneration process. Therefore, it is necessary to clarify the crosstalk among biomaterials, MSCs and macrophages.

In the early stage, we comprehensively investigated the direct regulatory effect of Ti surface topography on macrophage polarization and the consequent influence in osteogenic differentiation potential of MSCs by regulating macrophages [5]. In this study, we also verified the ability of the previously constructed nanoporous topography of TiO_2_ nanotubes with diameters of 80–100 nm to induce the osteogenic differentiation of bm-MSCs *in vitro*, and the results suggested that the expression of Runx2 and OCN in NT group was significantly elevated compared with the other groups. Runx2 is required for MSCs differentiation along the osteoblast lineage [22]. OCN is not only considered as a late-stage marker of osteogenesis, but also associated with insulin secretion [23]. Studies shows that high serum OCN concentration is beneficial for blood glucose control [24, 25]. So we assumed that the nanoporous topography of Ti may benefit the prognosis of diabetic bone implant materials and indirectly participating in the occurrence and development of diabetes by significantly up-regulating the OCN expression. The promoting effects on the osteogenic differentiation of bm-MSCs may be directly related to the increased roughness and hydrophilicity of the nanoporous topography.

Furthermore, Ti rods with different surface topographies were implanted into the cancellous bone of mice femurs, but the ability of nanoporous topography to induce osseointegration *in vivo* was inferior to the *in vitro* results. Histological observations revealed that a large number of inflammatory cells were concentrated around the implant in NT group, including M1-polarized macrophages, which was similar to PT group. The result that the osteogenesis abilities induced by the nanoporous topography *in vivo* and *in vitro* were not identical was consistent with our previous studies, and the changes in macrophage functions are critical for the implant prognosis. In addition to the direct regulation influence of nano-topography on macrophage polarization confirmed by previous studies, the immunomodulatory effects of MSCs could not be ignored.

The immunomodulatory effects of MSCs have attracted considerable attention in recent years [14]. The immunomodulatory effects of MSCs are plastic, and paracrine function is an important way to exert immune regulation. Under normal circumstances, MSCs can secrete inflammatory regulatory factors and pro-angiogenic factors after being stimulated by inflammation [26]. Therefore, stem-cell therapy has been extensively studied in the treatment of various immune inflammatory diseases, including colitis [27–29]. EVs, which are important for information communication between cells, contribute largely to maintaining tissue homeostasis [30]. As nanoscale extracellular lipid bilayer vesicles, exosomes can transport a large number of bioactive molecules to target cells, participating in basic physiological processes including immune response [31] and promoting pathological processes including inflammation [32]. The immunomodulatory function of MSCs can be realized by paracrine effects. Exosomes, as important components of paracrine products of MSCs, are affected by plenty of extracellular factors; among them, the properties of cell culture substrate are the key influencing factors. Studies have shown that the micro-patterned topography of Ti could promote exosome biogenesis and secretion, which influence the osteogenesis process [33, 34]. This is similar to the direction of our attention. After exosomes are released, they could be involved in the immunomodulatory process [26]. Studies have revealed that exosomes secreted by human umbilical cord MSCs can inhibit the overactivation of monocytes and macrophages, thus alleviating the acute liver injury [35]. In addition, exosomes derived from adipose MSCs are prone to induce M2 macrophage polarization [36]. Exosomes derived from MSCs can also achieve pulmonary function recovery through macrophage immune regulation [37]. In conclusion, a growing body of evidence suggests that exosomes might be involved in the regulation of macrophage functions. However, the paracrine function of MSCs could be affected by the microenvironment, for example, in the peri-implant microenvironment, bm-MSCs may be affected by the nanoporous topography of Ti, thus altering its paracrine function. Li showed that the topographical cues of materials could modulate paracrine functions of MSCs [38]. Therefore, we aimed to further investigate the impact of the nanoporous topography, which is extremely beneficial to osteogenesis *in vitro*, on the immune regulation of bm-MSCs. Through indirect co-culturing, we clarified that paracrine products of bm-MSCs can promote M1 polarization of macrophages under the induction of nanoporous topography with 80–100 nm tube diameters, but whether this induction effect is caused by exosomes remains to be verified.

By inhibiting the secretion of exosomes using GW4869 and repeating the above experimental process, we found that the effect of bm-MSCs induced by nanoporous topography on the M1 polarization of macrophages was significantly weakened, whereas the expression of M2 polarization markers was hardly affected, which suggested that the exosomes may be the key role that regulates macrophage M1 polarization through paracrine pathway of bm-MSCs. Compared with the blank control, we found that after the secretion of exosomes was blocked, the effect of bm-MSCs on inducing M1 polarization of macrophages in PT group was also significantly weakened, suggesting that compared with the ordinary culture plates, the matrix stiffness and hardness of Ti could be sensitively recognized by bm-MSCs, thus inducing changes in paracrine regulatory functions. Sridharan showed that the substrate stiffness could regulate the communication and immunomodulatory effects of MSCs on macrophages [39]. Therefore, compared with the Ti substrate, the nanoporous topography on Ti might play a far stronger role in the immune regulation of bm-MSCs. Exosomes were obtained via gradient centrifugation, and after the characterization of exosomes in different groups, we charified the independent effects of exosomes from different bm-MSCs on macrophage polarization. The results confirmed that under the induction of nanoporous topography, bm-MSCs may secrete exosomes containing special bioactive molecules, and the exosomes can be uptaken by macrophages, thus having immunomodulatory effects on macrophage M1 polarization.

The results demonstrate that, induced by the physical topography of Ti surface, bm-MSCs could exert an immunomodulatory effect on macrophage polarization through the exosome-mediated paracrine pathway. The finding complements our previous studies on the communications between bm-MSCs and macrophages in the peri-implant microenvironment, which is highly innovative and has directive significance in the MSC treatment with exosome targeting and the improvement of implant prognosis. However, further research should be performed to determine the key bioactive molecules in exosomes, which can regulate macrophage M1 polarization, and to charify how the nanoporous topography could regulate the immunomodulatory effects of bm-MSCs.

## Conclusion

This study clarified the complex immunomodulatory effects of bm-MSCs on macrophage M1 polarization, suggesting that in the absence of inflammatory stimuli, bm-MSCs can play a pro-inflammatory role under the influence of nanoporous topography with 80–100 nm tube diameters, supplementing the crosstalk among biomaterials, bm-MSCs and macrophages in the peri-implant microenvironment. Together, the biophysical cues such as topography that are presented by biomaterials can be tuned to play active roles in interactions between MSCs and macrophages, which in turn regulate the post-implantation response.

## References

[1] Ma SQ , Li XW, Hu H, Ma XY, Zhao ZZ, Deng S, Wang J, Zhang LY, Wu CX, Liu ZH, Wang YL. Synergetic osteogenesis of extracellular vesicles and loading RGD colonized on 3D-printed titanium implants. Biomater Sci 2022;17:4773–84. 10.1039/d2bm00725h 35849688

[2] Tasso R , Fais F, Reverberi D, Tortelli F, Cancedda R. The recruitment of two consecutive and different waves of host stem/progenitor cells during the development of tissue-engineered bone in a murine model. Biomaterials 2010;31:2121–9. 20004968 10.1016/j.biomaterials.2009.11.064

[3] Zhang JK , Zhao CC, Sheng RL, Lin KL, Wang XD, Zhang SL. Construction of a hierarchical micro-/submicro-/nanostructured 3D-printed Ti6Al4V surface feature to promote osteogenesis: involvement of Sema7A through the ITGB1/FAK/ERK signaling pathway. ACS Appl Mater Interfaces 2022;27:30571–81. 10.1021/acsami.2c06454 35776897

[4] Nakamoto ML , Forró C, Zhang W, Tsai CT, Cui BX. Expansion microscopy for imaging the cell-material interface. ACS Nano 2022;16:7559–71. 35533401 10.1021/acsnano.1c11015 PMC9879138

[5] Wang JJ , Meng FH, Song W, Jin JY, Ma QL, Fei DD, Fang L, Chen LH, Wang QT, Zhang YM. Nanostructured titanium regulates osseointegration via influencing macrophage polarization in the osteogenic environment. IJN 2018;13:4029–43. 30022825 10.2147/IJN.S163956 PMC6045901

[6] Ma QL , Fang L, Jiang N, Zhang L, Wang Y, Zhang YM, Chen LH. Bone mesenchymal stem cell secretion of sRANKL/OPG/M-CSF in response to macrophage-mediated inflammatory response influences osteogenesis on nanostructured Ti surfaces. Biomaterials 2018;154:234–47. 29144982 10.1016/j.biomaterials.2017.11.003

[7] Ma QL , Zhao LZ, Liu RR, Jin BQ, Song W, Wang Y, Zhang YS, Chen LH, Zhang YM. Improved implant osseointegration of a nanostructured titanium surface via mediation of macrophage polarization. Biomaterials 2014;37:9853–67. 10.1016/j.biomaterials.2014.08.025 25201737

[8] Pérez S , Rius-Pérez S. Macrophage polarization and reprogramming in acute inflammation: a redox perspective. Antioxidants (Basel*)* 2022;7:1394. 10.3390/antiox11071394 PMC9311967 35883885

[9] Guo WR , Wu XP, Wei WY, Wang YF, Dai HL. Mesoporous hollow FeO nanoparticles regulate the behavior of neuro-associated cells through induction of macrophage polarization in an alternating magnetic field. J Mater Chem B 2022;29:5633–43. 10.1039/d2tb00527a 35816162

[10] Yu JW , Lin YF, Wang GW, Song JL, Hayat U, Liu C, Raza A, Huang XY, Lin HD, Wang JY. Zein-induced immune response and modulation by size, pore structure and drug-loading: application for sciatic nerve regeneration. Acta Biomater 2022;140:289–301. 34843952 10.1016/j.actbio.2021.11.035

[11] Wang XQ , Shah FA, Vazirisani F, Johansson A, Palmquist A, Omar O, Ekström K, Thomsen P. Exosomes influence the behavior of human mesenchymal stem cells on titanium surfaces. Biomaterials 2020;230:119571. 31753474 10.1016/j.biomaterials.2019.119571

[12] Chen LW , Tredget EE, Wu YG, Wu YJ. Paracrine factors of mesenchymal stem cells recruit macrophages and endothelial lineage cells and enhance wound healing. PLoS ONE 2008;4:e1886. 10.1371/journal.pone.0001886 PMC2270908 18382669

[13] Gao S , Mao F, Zhang B, Zhang L, Zhang X, Wang M, Yang YM, Yang TT, Zhang J, Zhu W, Qian H, Xu WR. Mouse bone marrow-derived mesenchymal stem cells induce macrophage M2 polarization through the nuclear factor-kappaB and signal transducer and activator of transcription 3 pathways. Exp Biol Med (Maywood*)* 2014;3:366–75. 10.1177/1535370213518169 24500984

[14] Psaroudis RT , Singh U, Lora M, Jeon P, Boursiquot A, Stochaj U, Langlais D, Colmegna I. CD26 is a senescence marker associated with reduced immunopotency of human adipose tissue-derived multipotent mesenchymal stromal cells. Stem Cell Res Ther 2022;1:358. 10.1186/s13287-022-03026-4 PMC9327293 35883188

[15] Boland L , Bitterlich LM, Hogan AE, Ankrum JA, English K. Translating MSC therapy in the age of obesity. Front Immunol 2022;13:943333. 35860241 10.3389/fimmu.2022.943333 PMC9289617

[16] Zhao XG , Zhao YH, Sun X, Xing Y, Wang X, Yang Q. Immunomodulation of MSCs and MSC-derived extracellular vesicles in osteoarthritis. Front Bioeng Biotechnol 2020;8:575057. 33251195 10.3389/fbioe.2020.575057 PMC7673418

[17] Corrado C , Raimondo S, Chiesi A, Ciccia F, Leo GD, Alessandro R. Exosomes as intercellular signaling organelles involved in health and disease: basic science and clinical applications. Int J Mol Sci 2013;3:5338–66. 10.3390/ijms14035338 PMC3634447 23466882

[18] Kastelowitz N , Yin H. Exosomes and microvesicles: identification and targeting by particle size and lipid chemical probes. Chembiochem 2014;7:923–8. 10.1002/cbic.201400043 PMC4098878 24740901

[19] Shen B , Liu J, Zhang F, Wang Y, Qin Y, Zhou ZH, Qiu JX, Fang Y. CCR2 positive exosome released by mesenchymal stem cells suppresses macrophage functions and alleviates ischemia/reperfusion-induced renal injury. Stem Cells Int 2016;2016:1–9. 10.1155/2016/1240301 PMC5098097 27843457

[20] Zhao JX , Li XL, Hu JX, Chen F, Qiao SH, Sun X, Gao L, Xie J, Xu B. Mesenchymal stromal cell-derived exosomes attenuates myocardial ischemia-reperfusion injury through miR-182-regulated macrophage polarization. Cardiovasc Res 2019;7:1205–16. 10.1093/cvr/cvz040 PMC6529919 30753344

[21] Xin LB , Lin XN, Zhou F, Li C, Wang XF, Yu HY, Pan YB, Fei HY, Ma L, Zhang SY. A scaffold laden with mesenchymal stem cell-derived exosomes for promoting endometrium regeneration and fertility restoration through macrophage immunomodulation. Acta Biomater 2020;113:252–66. 32574858 10.1016/j.actbio.2020.06.029

[22] Li XD , Cui QJ, Kao CH, Wang GJ, Balian G. Lovastatin inhibits adipogenic and stimulates osteogenic differentiation by suppressing PPARgamma2 and increasing Cbfa1/Runx2 expression in bone marrow mesenchymal cell cultures. Bone 2003;4:652–9. 10.1016/s8756-3282(03)00239-4 14555271

[23] Shao J , Wang Z, Yang TY, Ying H, Zhang Y, Liu SY. Bone regulates glucose metabolism as an endocrine organ through osteocalcin. Internet J Endocrinol 2015;2015:967673. 10.1155/2015/967673 PMC4383405 25873961

[24] Wang Y-J , Jin C-H, Ke J-F, Wang J-W, Ma Y-L, Lu J-X, Li M-F, Li L-X. Decreased serum osteocalcin is an independent risk factor for metabolic dysfunction-associated fatty liver disease in type 2 diabetes. Dmso 2022;15:3717–28. 10.2147/DMSO.S389794 PMC9719286 36471670

[25] Lei H , Liu J, Wang W, Yang X, Feng Z, Zang P, Lu B, Shao J. Association between osteocalcin, a pivotal marker of bone metabolism, and secretory function of islet beta cells and alpha cells in Chinese patients with type 2 diabetes mellitus: an observational study. Diabetol Metab Syndr 2022;14:160. 36307866 10.1186/s13098-022-00932-8 PMC9615358

[26] Shi YF , Wang Y, Li Q, Liu KL, Hou JQ, Shao CS, Wang Y. Immunoregulatory mechanisms of mesenchymal stem and stromal cells in inflammatory diseases. Nat Rev Nephrol 2018;8:493–507. 10.1038/s41581-018-0023-5 29895977

[27] Heidari N , Abbasi-Kenarsari H, Namaki S, Baghaei K, Zali MR, Mirsanei Z, Hashemi SM. Regulation of the Th17/treg balance by human umbilical cord mesenchymal stem cell-derived exosomes protects against acute experimental colitis. Exp Cell Res 2022;30:113296. 10.1016/j.yexcr.2022.113296 35917844

[28] Aghayan HR , Salimian F, Abedini A, Ghazi SF, Yunesian M, Alavi-Moghadam S, Makarem J, Majidzadeh AK, Hatamkhani A, Moghri M, Danesh A, Haddad-Marandi MR, Sanati H, Abbasvandi F, Arjmand B, Azimi P, Ghavamzadeh A, Sarrami-Forooshani R. Human placenta-derived mesenchymal stem cells transplantation in patients with acute respiratory distress syndrome (ARDS) caused by COVID-19 (phase I clinical trial): safety profile assessment. Stem Cell Res Ther 2022;1:365. 10.1186/s13287-022-02953-6 PMC9330663 35902979

[29] Műzes G , Sipos F. Mesenchymal stem cell-derived secretome: a potential therapeutic option for autoimmune and immune-mediated inflammatory diseases. Cells 2022;15:2300. 10.3390/cells11152300 PMC9367576 35892597

[30] Pluchino S , Smith JA. Explicating exosomes: reclassifying the rising stars of intercellular communication. Cell 2019;2:225–7. 10.1016/j.cell.2019.03.020 30951665

[31] Robbins PD , Morelli AE. Regulation of immune responses by extracellular vesicles. Nat Rev Immunol 2014;14:195–208. 24566916 10.1038/nri3622 PMC4350779

[32] Kulshreshtha A , Ahmad T, Agrawal A, Ghosh B. Proinflammatory role of epithelial cell derived exosomes in allergic airway inflammation. J Allergy Clin Immunol 2013;4:1194–203.e1–14. 10.1016/j.jaci.2012.12.1565 23414598

[33] Hisey CL , Hearn JI, Hansford DJ, Blenkiron C, Chamley LW. Micropatterned growth surface topography affects extracellular vesicle production. Colloids Surf B Biointerfaces 2021;203:111772. 33894649 10.1016/j.colsurfb.2021.111772

[34] Zhang Z , Xu R, Yang Y, Liang C, Yu X, Liu Y, Wang T, Yu Y, Deng F. Micro/nano-textured hierarchical titanium topography promotes exosome biogenesis and secretion to improve osseointegration. J Nanobiotechnol 2021;19:78. 10.1186/s12951-021-00826-3 PMC7980346 33741002

[35] Shao MY , Xu Q, Wu ZR, Chen YW, Shu YK, Cao XY, Chen ML, Zhang B, Zhou YJ, Yao R, Shi YJ, Bu H. Exosomes derived from human umbilical cord mesenchymal stem cells ameliorate IL-6-induced acute liver injury through miR-455-3p. Stem Cell Res Ther 2020;1:37. 10.1186/s13287-020-1550-0 PMC6979401 31973730

[36] Heo JS , Choi Y, Kim HO. Adipose-derived mesenchymal stem cells promote M2 macrophage phenotype through exosomes. Stem Cells Int 2019;2019:7921760. 31781246 10.1155/2019/7921760 PMC6875419

[37] Willis GR , Fernandez-Gonzalez A, Anastas J, Vitali SH, Liu XL, Ericsson M, Kwong A, Mitsialis SA, Kourembanas S. Mesenchymal stromal cell exosomes ameliorate experimental bronchopulmonary dysplasia and restore lung function through macrophage immunomodulation. Am J Respir Crit Care Med 2018;1:104–16. 10.1164/rccm.201705-0925OC PMC5765387 28853608

[38] Li T , Ma HS, Ma HZ, Ma ZJ, Qiang L, Yang ZZ, Yang XX, Zhou XJ, Dai KR, Wang JW. Mussel-inspired nanostructures potentiate the immunomodulatory properties and angiogenesis of mesenchymal stem cells. ACS Appl Mater Interfaces 2019;19:17134–46. 10.1021/acsami.8b22017 31008578

[39] Sridharan R , Kelly DJ, O'Brien FJ. Substrate stiffness modulates the crosstalk between mesenchymal stem cells and macrophages. J Biomech Eng 2021;3. 10.1115/1.4048809 33067618

